# iDNAProt-ES: Identification of DNA-binding Proteins Using Evolutionary and Structural Features

**DOI:** 10.1038/s41598-017-14945-1

**Published:** 2017-11-02

**Authors:** Shahana Yasmin Chowdhury, Swakkhar Shatabda, Abdollah Dehzangi

**Affiliations:** 1 Department of Computer Science and Engineering, United International University, House 80, Road 8A, Dhanmondi, Dhaka 1209 Bangladesh; 2 Department of Computer Science, Morgan State University, Baltimore, Maryland United States

## Abstract

DNA-binding proteins play a very important role in the structural composition of the DNA. In addition, they regulate and effect various cellular processes like transcription, DNA replication, DNA recombination, repair and modification. The experimental methods used to identify DNA-binding proteins are expensive and time consuming and thus attracted researchers from computational field to address the problem. In this paper, we present iDNAProt-ES, a DNA-binding protein prediction method that utilizes both sequence based evolutionary and structure based features of proteins to identify their DNA-binding functionality. We used recursive feature elimination to extract an optimal set of features and train them using Support Vector Machine (SVM) with linear kernel to select the final model. Our proposed method significantly outperforms the existing state-of-the-art predictors on standard benchmark dataset. The accuracy of the predictor is 90.18% using jack knife test and 88.87% using 10-fold cross validation on the benchmark dataset. The accuracy of the predictor on the independent dataset is 80.64% which is also significantly better than the state-of-the-art methods. iDNAProt-ES is a novel prediction method that uses evolutionary and structural based features. We believe the superior performance of iDNAProt-ES will motivate the researchers to use this method to identify DNA-binding proteins. iDNAProt-ES is publicly available as a web server at: http://brl.uiu.ac.bd/iDNAProt-ES/.

## Introduction

DNA-binding proteins are those proteins that bind and interact with DNA. DNA-binding proteins play important role in the structural composition of the DNA and in gene regulations. Non-specific structural proteins often help to organize and compact the chromosomal DNA. The other important role is to regulate and effect various cellular processes like transcription, DNA replication, DNA recombination, repair and modification. These proteins in their independently folded domains have at least one structural motif and have affinity to DNA^1^. DNA-binding proteins or ligands have many important applications as antibiotics, drugs, steroids for various biological effects and in bio-physical, bio-chemical and biological studies of DNA^2^.

Many experimental methods are being used to identify DNA-binding proteins: filter binding assays^3^, genetic analysis^4^, X-ray crystallography^5^, chromatin immunoprecipitation on microarrays^6^, NMR^7,8^ etc. However, these experimental methods are costly and time consuming^9^. Therefore, there is a growing interest in finding new computational methods to replace experimental methods to identify DNA-binding proteins. Moreover, the number of newly discovered protein sequences has been increasing extremely fast due to the advent of modern protein sequencing technologies. For example, in 1986 the Swiss-Prot^10^ database contained only 3,939 protein sequence entries, but now the number has jumped to 88,032,926 according to the release 2017_07 of July, 5, 2017 by the UniProtKB/Swiss-Prot (http://web.expasy.org/docs/relnotes/relstat.html). It means that the number of protein sequence entries is now thausands times more than the number from about 25 years ago. Facing the flood of new protein sequences generated in the post genomic age, it is highly desired to develop automated computational prediction approaches for rapidly and effectively identifying and characterizing DNA-binding proteins.

Computational methods that have been used to predict the DNA-binding proteins can be broadly categorized into two groups: structure based methods^11,12^ and sequence based methods^13–19^. In most of the cases, DNA-binding protein identification is formulated as a binary classification problem in the supervised learning setting. The sequence based methods are built depending only on the sequence based information extracted from the training data where structure based methods also exploits structure based features. In^20^, structural motifs and electrostatic potentials were used to predict DNA-binding proteins. DNA-binding domain hunter (DBD-Hunter)^21^ was proposed to identify DNA-binding proteins using structure comparison and evaluation of a statistical potential derived from the interactions between DNA base pairs and protein residues. The iDBPs server was proposed in^22^ used global features like average surface electrostatic potential, the dipole moment and cluster-based amino acid conservation patterns. Low resolution *α*-carbon only models generated by TASSER^23^ to predict DNA-binding proteins in^24^. One of the major difficulties in structure based methods is that the structure of most of the proteins are unknown. However, structural information like presence of motifs and other information is very crucial in DNA recognition of binding proteins. Therefore, we hypothesize that even partial information of the protein structure could play very important role in identifying their function of binding DNA.

Many machine learning algorithms are applied to solve this problem in the literature. Among them are: Logistic Regression^24^, Hidden Markov Models^20^, Random Forest^22,25,26^, Artificial Neural Network^27^, Support Vector Machines^14,28^, Naive Bayes classifier^15^ etc. A number of softwares, web-servers and prediction methods are available in the literature for DNA-binding protein prediction. Among them are: DNABinder^28^, DNA-Prot^25^, iDNA-Prot^26^, iDNA-Prot|dis^13^, DBPPred^15^, iDNAPro-PseAAC^14^, PseDNA-Pro^29^, Kmer1 + ACC^30^, Local-DPP^16^, etc. Kumar *et al*.^28^ used evolutionary information from PSSM profiles with support vector machines and established a web-server called DNABinder. They compared the effectiveness of the PSSM based features with amino acid composition, di-peptide composition and 4-parts amino acid compositions as features.

DNA-Prot is another software proposed in^25^. They used amino acid composition, physio-chemical properties and secondary structure information as features and trained their model using a Random Forest classifier. Lin *et al*.^26^ presented a web-server named iDNA-Prot where they used grey model to incorporate amino acid sequence as features into the general form of pseudo amino acid composition and trained their model using Random Forest classifier. Amino acid distance-pair coupling information and the amino acid reduced alphabet profile was incorporated into the general form of pseudo amino acid composition^31^ by Liu *et al*.^13^. They also offered a freely available web-server called iDNA-Prot|dis. On of the most successful prediction method so far is DBPPred proposed in^15^. They used a wrapper based best first feature selection technique to select optimal set of features. They used features based on amino acid composition, PSSM scores, secondary structures and relative solvent accessibility and trained their model using Random Forest and Gaussian Naive Bayesian classifiers.

Liu *et al*.^14^ presented iDNAPro-PseAAC as a web server. They used evolutionary information as their input features. They used profile-based protein representation and selected a set of 23 optimal features using Linear Discriminant Analysis (LDA). Their model was trained using Support Vector Machine (SVM) classifier. Kmer composition and auto-cross covariance transformation was used in^30^ in a subsequent work. Their method trained by SVM is known as Kmer1 + ACC in the literature. They also developed another server called PseDNA-Pro^29^. PseDNA-Pro used amino acid composition, pseudo amino acid composition and physicochemical distance transformation based features to train their model. Wei *et al*. proposed Local-DPP^16^ by using Random Forest classifier on local pseudo position specific scoring matrix features. Among other recent works are SVM-PSSM-DT^32^, PNImodeler^33^, CNNsite^34^, BindUP^35^, etc.

One of the most important but also most difficult problems in computational biology is how to express a biological sequence with a discrete model or a vector, yet still keep considerable sequence-order information or key pattern characteristic. This is because all the existing machine-learning algorithms can only handle vector but not sequence samples, as elucidated in a recent review^36^. However, a vector defined in a discrete model may completely lose all the sequence-pattern information. To avoid completely losing the sequence-pattern information for proteins, the pseudo amino acid composition or PseAAC^37^ was proposed. Ever since then, the concept of PseAAC has been rapidly and widely penetrated into nearly all the areas of computational proteomics^38,39^. Because it has been widely and increasingly used, recently three powerful open access soft-wares, called ‘PseAAC-Builder’, ‘propy’, and ‘PseAAC-General’, were established: the former two are for generating various modes of Chou’s special PseAAC; while the 3rd one for those of Chou’s general PseAAC, including not only all the special modes of feature vectors for proteins but also the higher level feature vectors such as “Functional Domain” mode, “Gene Ontology” mode, and “Sequential Evolution” or “PSSM” mode. Encouraged by the successes of using PseAAC to deal with protein or peptide sequences, four web-servers called ‘PseKNC’, ‘PseKNC-General’, ‘repDNA’, and ‘repRNA’ were developed for generating various feature vectors for DNA/RNA sequences as well. Particularly, recently a very powerful web-server called Pse-in-One^40^ has been established that can be used to generate any desired feature vectors for protein or peptides and DNA or RNA sequences according to the need of users’ studies. In the current study, we are to use 14 different modes of the general PseAAC derived from evolutionary and structural informations to identify DNA-binding proteins.

As done in a series of recent publications^41–48^ in compliance with Chou’s 5-step rule, to establish a really useful sequence-based statistical predictor for a biological system, we should follow the following five guidelines: (a) construct or select a valid benchmark dataset to train and test the predictor; (b) formulate the biological sequence samples with an effective mathematical expression that can truly reflect their intrinsic correlation with the target to be predicted; (c) introduce or develop a powerful algorithm to operate the prediction; (d) properly perform cross-validation tests to objectively evaluate the anticipated accuracy of the predictor; (e) establish a user-friendly web-server for the predictor that is accessible to the public. In this paper, we propose iDNAProt-ES, **i**dentification of **DNA**-binding **Prot**eins using **E**volutionary and **S**tructure Features. In our proposed method, a number of novel features have been derived from sequence based evolutionary information and structural information of a given protein to train a SVM classifier with linear kernel. We used recursive feature elimination technique to reduce the number of features and to derive an optimal set of features for DNA-binding protein prediction. We have tested our method on standard benchmark datasets. Experimental results show that iDNAProt-ES significantly outperforms other state-of-the-art methods found in the literature and thus have potentials to be used as a DNA-binding protein prediction tool.

## Results and Discussion

In this section, we present the results of the experiments that were carried out in this study. All the methods were implemented in Python language using Python3.4 version and Scikit-learn library^49^ of Python was used for the implementation of machine learning algorithms. All experiments were conducted on a Computing Machine provided by CITS, United International University. Each of the experiments were carried 50 times and only the average is reported as results.

### Comparison With Other Methods

To compare the performance of our predictor iDNAProt-ES with the state-of-the-art algorithms found in the literature, we first used the benchmark dataset. using this dataset, we performed jack knife test and report accuracy, sensitivity, specificity, MCC and auROC values in Table 1. We compare the results achieved by iDNAProt-ES with previous state-of-the-art methods found in the literature including: DNABinder^28^, DNA-Prot^25^, iDNA-Prot^26^, iDNA-Prot|dis^13^, DBPPred^15^, iDNAPro-PseAAC^14^, PseDNA-Pro^29^, Kmer1 + ACC^30^ and Local-DPP^16^. The results reported in this paper for these methods are taken from^14,16^.

**Table 1:** Comparison of performance of the proposed method with other state-of-the-art predictors using jack knife test on the benchmark dataset.

Method	Accuracy	Sensitivity	Specificity	MCC	auROC
iDNAPro-PseAAC	76.76%	0.7562	0.7745	0.53	0.8392
DNAbinder (dimension 21)	73.95%	0.6857	0.7909	0.48	0.8140
DNAbinder (dimension 400)	73.58%	0.6647	0.8036	0.47	0.8150
DNA-Prot	72.55%	0.8267	0.5976	0.44	0.7890
iDNA-Prot	75.40%	0.8381	0.6473	0.50	0.7610
iDNA-Prot|dis	77.30%	0.7940	0.7527	0.54	0.8310
PseDNA-Pro	76.55%	0.7961	0.7363	0.53	—
Kmer1 + ACC	75.23%	0.7676	0.7376	0.50	0.8280
Local-DPP	79.20%	0.8400	0.7450	0.59	—
iDNAProt-ES	**90**.**18%**	**0**.**9038**	**0**.**9000**	**0**.**8036**	**0**.**9412**

The best values in Table 1 are shown in bold faced font. For the benchmark dataset our method iDNAProt-ES significantly outperforms the previous state-of-th-art in terms of all the evaluation metrics used. Accuracy of iDNAProt-ES is 90.18% compared to the previous best 79.20% by Local-DPP^16^. The higher MCC value and auROC also depicts the effective ness of our method.

To assess the performance and generality of iDNAProt-ES further, we applied it on the independent dataset introduced in^15^. Here, we used the same model trained using iDNAProt-ES on the benchmark dataset and tested using the independent dataset. We report the performance metrics in Table 2 for the independent dataset. Here too the best values are shown in bold faced font. We could notice that our algorithm is showing better performance in terms of accuracy and auROC compared to the other state-of-the-art algorithms. However, the sensitivity, specificity and MCC values are not the best, but comparable to the other methods. Although we demonstrate consistant prediction performance enhancement for both train and test benchmarks, yet the improvement achieved on the train set is larger than the test set. The main reasons for such phenomena are because of feature selection and parameter tuning steps that are conducted on the train set. Despite we made sure that we separate a validation set for those tasks, still it is possible that the tuned parameters are more homogeneous to samples in the train set. However, repeating the enhancement on the independent test benchmark support the generality of our proposed method.

**Table 2:** Comparison of performance of the proposed method with other state-of-the-art predictors on the independent dataset.

Method	Accuracy	Sensitivity	Specificity	MCC	auROC
iDNAPro-PseAAC	69.89%	0.7741	0.6237	0.402	0.7754
iDNA-Prot	67.20%	0.6770	0.6670	0.344	—
DNA-Prot	61.80%	0.6990	0.5380	0.240	—
DNAbinder	60.80%	0.5700	0.6450	0.216	0.6070
DNABIND	67.70%	0.6670	0.6880	0.355	0.6940
DNA-Threader	59.70%	0.2370	**0**.**9570**	0.279	—
DBPPred	76.90%	0.7960	0.7420	0.538	0.7910
iDNA-Prot|dis	72.00%	0.7950	0.6450	0.445	0.7860
Kmer1 + ACC	70.96%	0.8279	0.5913	0.431	0.7520
Local-DPP	79.00%	**0**.**9250**	0.6560	**0**.**625**	—
iDNAProt-ES	**80**.**64%**	0.8131	0.8000	0.6130	**0**.**8434**

### Effect of Feature Selection

**Figure 1:**
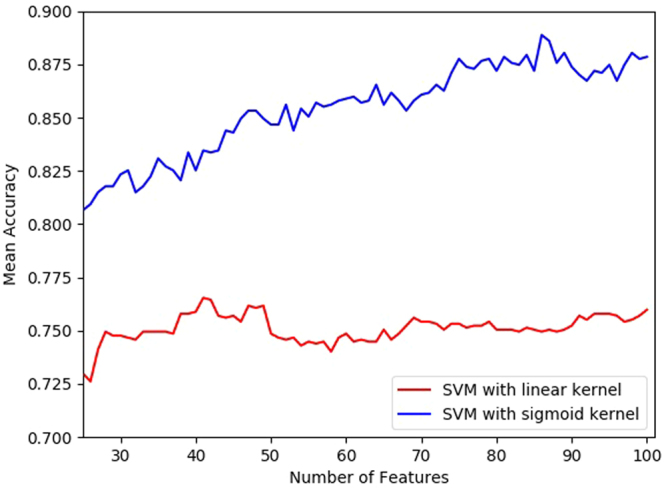
Effect of number of features selected on the accuracy on the benchmark dataset.

In this section, we show the effect of the feature selection algorithm that we used. For this experiment we used 10-fold cross validation on both of the datasets to find the optimal set of features using recursive feature elimination technique. We varied the number of features from 25 $\cdots$ 100 using the recursive feature elimination technique for two SVM kernels: sigmoid and linear. The highest accuracy was found when the number of reduced features were set to 86. Figure 1 shows the plot of accuracy against the number of reduced features using recursive feature selection algorithm using two classifiers. The list of selected features are provided in Suplementary file 1.

**Figure 2:**
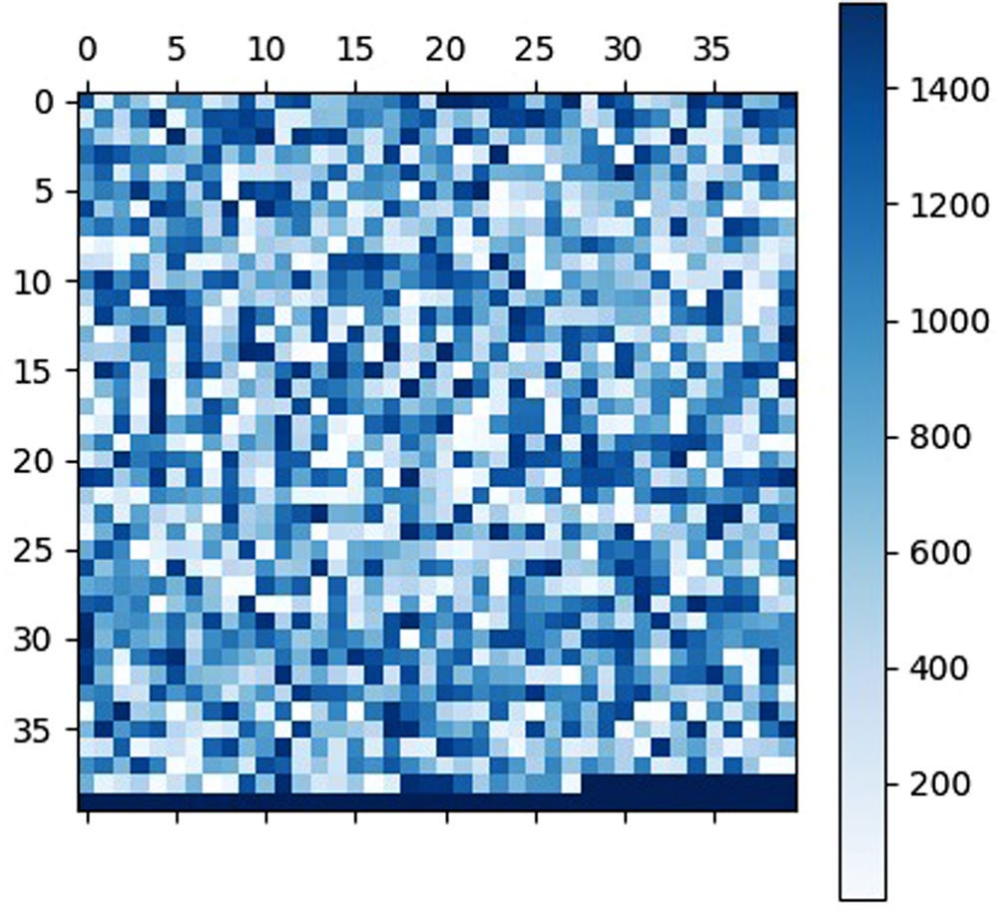
Color map showing the importance or ranking of the features on the benchmark dataset.

Color map of the rankings of the features as ranked by the RFE algorithm is given in Fig. 2. This color map depicts the distribution of selected features over all the features. Selected features include Dubchuck features, PSSM bigram, PSSM Auto-Covariance, PSSM 1-lead bigram and PSSM segmented distribution from the evolutionary group of features extracted for PSSM and the rest of the features were structural features generated by SPIDER2. It reveals the importance of both type of features: evolutionary and structural. A list of selected features is given in the supporting information.

We then compared the performance of this feature selection technique with other feature selection techniques: tree based method^50^ and randomized sparse elimination^51,52^ and with no feature elimination. We performed 10-fold cross validation for these experiments too and applied different feature elimination techniques on the benchmark dataset and report the results in Table 3.

**Table 3:** Comparison of performance of different feature selection methods on the benchmark dataset using 10-fold cross validation.

Method	Accuracy	Sensitivity	Specificity	MCC	auROC	auPR
RFE	**88**.**87%**	**0**.**8945**	**0**.**8826**	**0**.**7788**	**0**.**9391**	**0**.**8828**
Tree Based Method	70.93%	0.7627	0.6480	0.4196	0.7775	0.6470
Sparse Elimination	75.98%	0.7727	0.7461	0.5210	0.8308	0.7464
No Feature Selection	74.01%	0.7581	0.7211	0.4835	0.8224	0.7242

**Figure 3:**
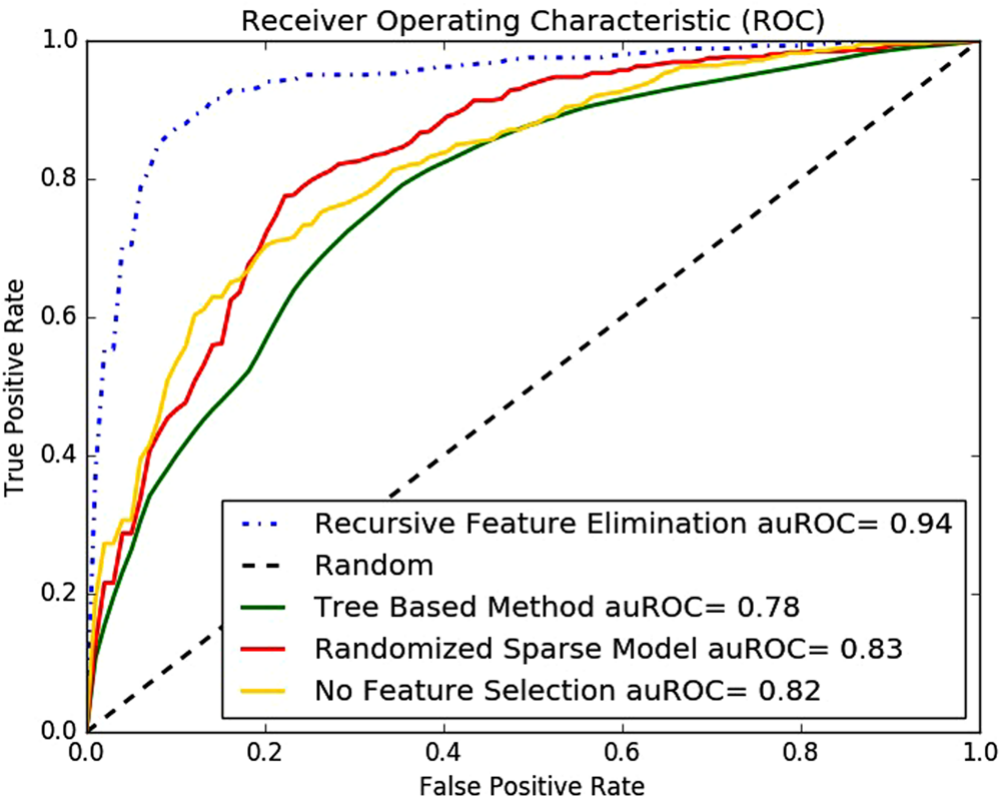
Receiver Operating Characteristic (ROC) curve of different feature selection methods on the benchmark dataset.

Here too, we show the best values achieved in bold faced fonts. We could easily note that recursive feature elimination technique was the best among the feature elimination techniques that were used in the experiments. We also show the Receiver Operating Curve (ROC) for each of these methods for the benchmark dataset in Fig. 3.

### Effect of Classifier Selection

To justify the classifier selection for our algorithm, we ran another set of experiments on the benchmark dataset using 10-fold cross validation. Several classifiers were tested in the experiments: SVM with linear kernel, SVM with Radial Basis Function (RBF) kernel, SVM with sigmoid kernel, Random Forest Classifier, Naive Bayes Classifier and Logistic Regression Classifier. The results achieved in these experiments are shown in Table 4.

**Table 4:** Comparison of performance of different Classifiers on the benchmark dataset using 10-fold cross validation.

Classifier	Accuracy	Sensitivity	Specificity	MCC	auROC	auPR
SVM (linear kernel)	**88**.**87%**	**0**.**8945**	**0**.**8826**	**0**.**7788**	**0**.**9391**	**0**.**8828**
SVM (rbf kernel)	81.96%	0.8309	0.8076	0.6415	0.8866	0.8117
SVM (sigmoid kernel)	56.07%	0.5672	0.5538	0.1218	0.6010	0.5527
Random Forest	70.56%	0.7636	0.6442	0.4107	0.7881	0.6451
Naive Bayes	61.58%	0.7545	0.4692	0.2362	0.7005	0.4726
Logistic Regression	86.72%	0.8800	0.8538	0.7359	0.9359	0.8567

**Figure 4:**
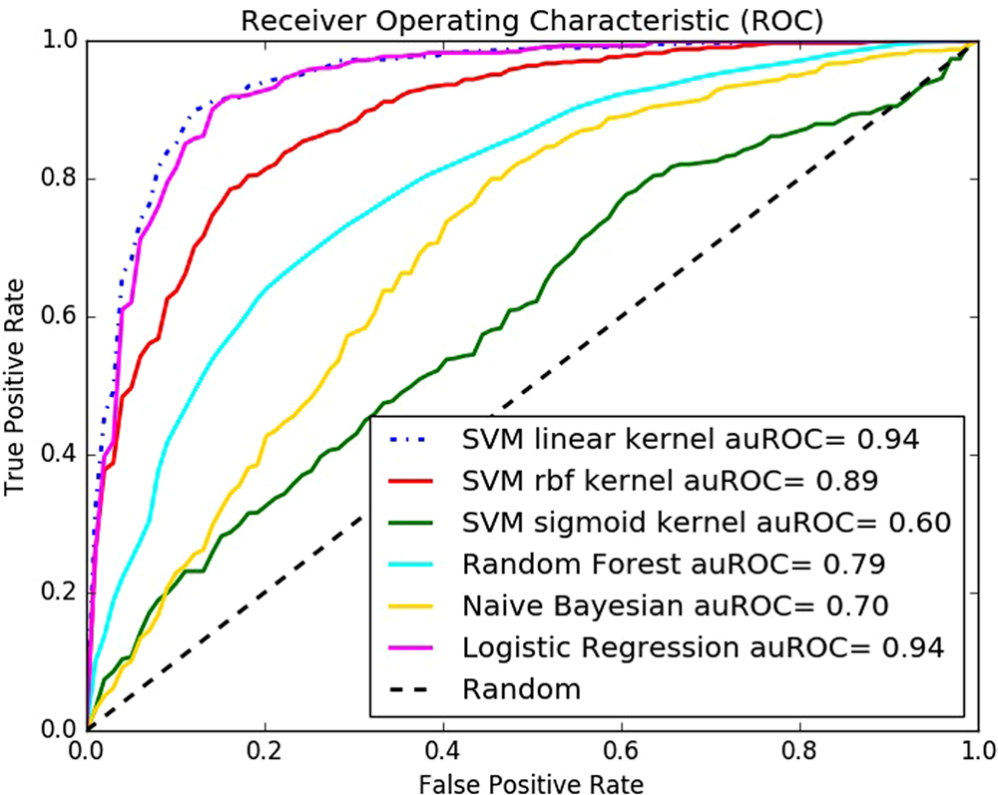
Receiver Operating Characteristic (ROC) curve of different classifiers for the benchmark dataset.

The best values in Table 4 are shown in bold faced fonts. We could see the SVM classifier with linear kernel outperformed all other classifiers. The closest competitor to linear kernel was the logistics regression classifier and the SVM with RBF kernel. We also show the ROC curve for this experiment in Fig. 4.

### Web Server Implementation

**Figure 5:**
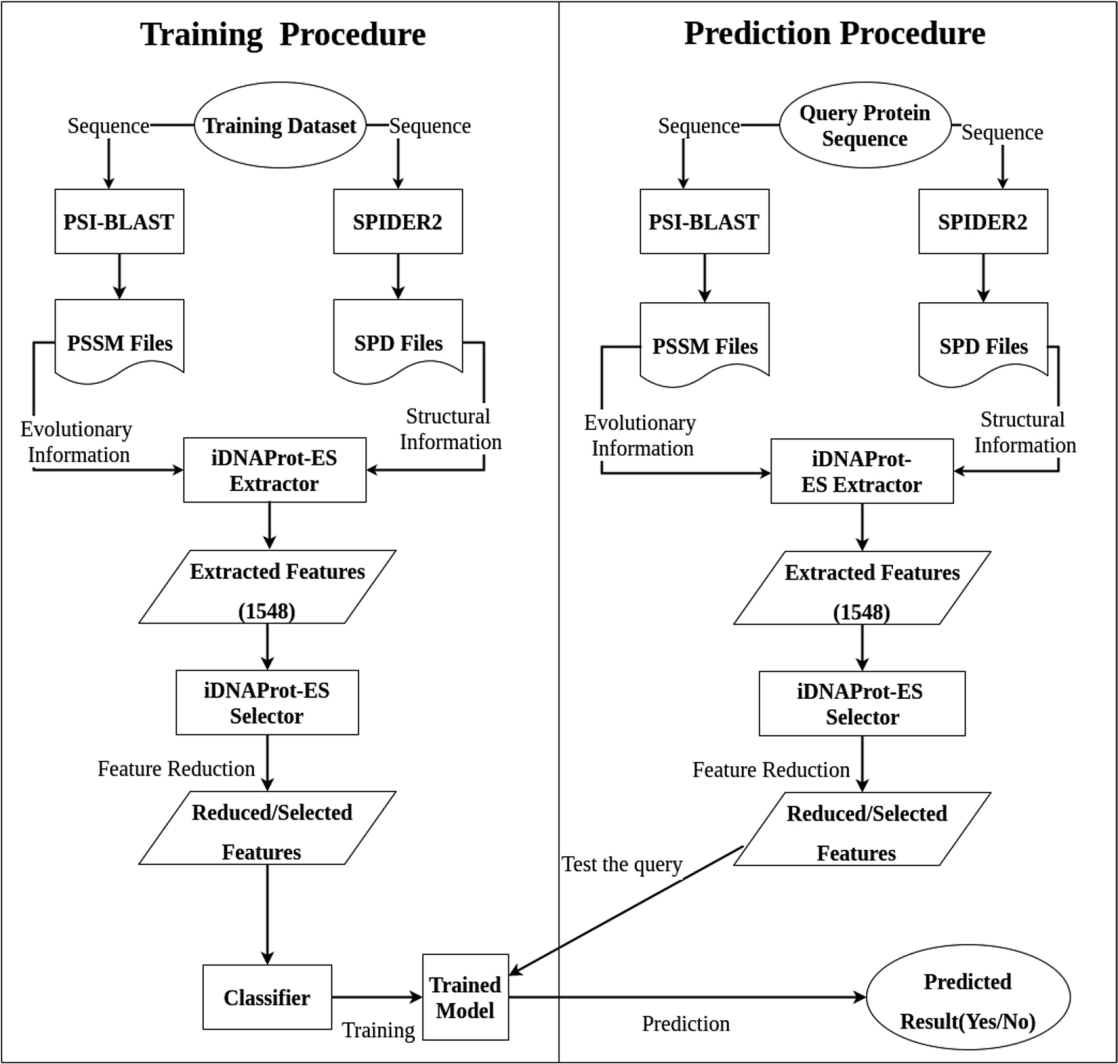
System flow diagram of iDNAProt-ES showing the training and prediction procedure as flowchart.

**Figure 6:**
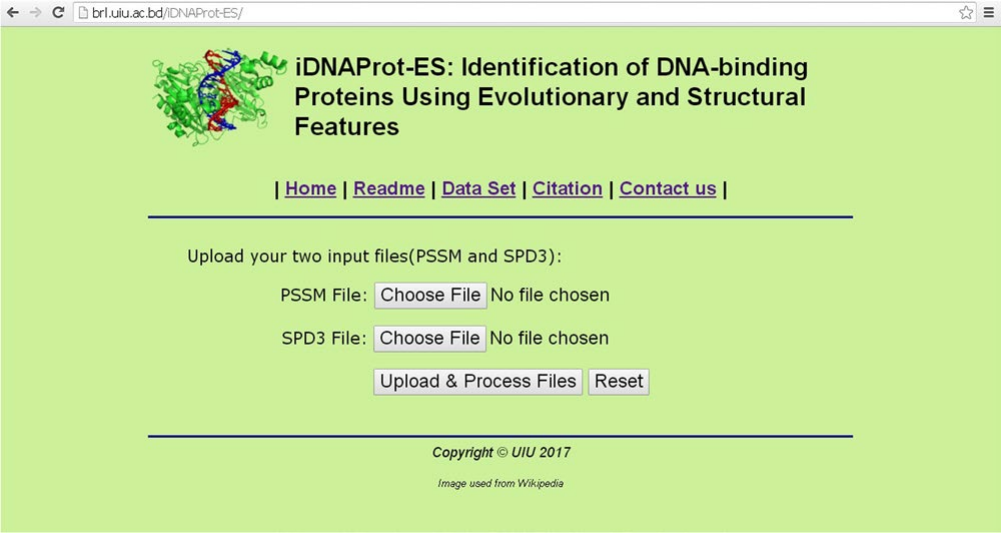
Screen shot of Web-Server homepage.

To make the predictor iDNAProt-ES freely available for use and test we implemented a web server. This web application is freely available to use at: http://brl.uiu.ac.bd/iDNAProt-ES/. This is a very easy to use website and the model here is trained using the benchmark dataset. To use this site for identification of DNA-binding proteins, one has to provide two input files: PSSM file generated by PSI-BLAST^53^ and a SPD file generated by SPIDER2^54^. After these files are uploaded iDNAProt-ES, will extract features and follow a similar procedure as shown in Fig. 5. A detail guideline is provided in the website to use the predictor. A screen-shot of the web application is given in Fig. 6. As pointed out in^39^ and demonstrated in a series of recent publications^41–48,55^, user-friendly and publicly accessible web-servers represent the future direction for developing practically more useful prediction methods and enhance their impact^39^, we shall make efforts to assure the iDNAProt-ES server is always in the normal working state.

## Materials and Methods

To establish a novel feature set and good predictor we first collected two benchmark datasets. We then extracted features from the data sets which are able to discriminate the DNA-binding proteins, develop the list of reduced features from the global set of features which can contribute to improve prediction accuracy of prediction, and selected and developed powerful classification algorithm to perform prediction. We finally performed cross-validation tests to evaluate the accuracy of predictor.

The framework of our proposed method iDNAProt-ES is depicted in the Fig. 5. There are two phases in the framework for prediction: *training phase* and *prediction phase*. In training phase, at first a training dataset is selected. Next, each protein sequence from the training dataset is then passed to the PSI-BLAST^53^ and SPIDER3^56^ softwares, that provide two output files PSSM and SPD3 respectively. PSSM file is responsible for evolutionary information and SPD3 is responsible for structural information. These two files are then passed to the iDNAProt-ES feature extractor, which extract 14 sets of features. These 14 feature sets contains total 1548 sub-features in total. Note that tools and application servers are available in the literature that extracts features from PSSM files^57^. Then all these extracted features (1548) from the feature extraction method are then passed to the iDNAProt-ES feature selector to reduced the features to improve the prediction accuracy. We can get the list of reduced feature set from this method which is provided in Supplementary file 1. The reduced features are used to train a model using SVM classifier and stored later for prediction.

In the prediction phase, iDNAProt-ES first a query protein sequence and passed to the PSI-BLAST and SPIDER3 to generate two output files PSSM and SPD3 respectively as similar to the training phase. These two files are then used by the feature extractor and feature selector of iDNAProt-ES. The reduced features are passed to the previously saved model in training phase to predict whether the protein is DNA-binding or not. These phase takes very little time compared to the training phase.

### Datasets

We require a set of reliable benchmark datasets in order to develop an effective predictor using suitable classification algorithm and feature set. Any dataset consists of positive and negative samples and can be formally denotes as following:

1 $${\mathbb{S}}={{\mathbb{S}}}^{+}\cup {{\mathbb{S}}}^{-}$$

Here ${{\mathbb{S}}}^{+}$ represents the set of positive instances or DNA-binding proteins and ${{\mathbb{S}}}^{-}$ denotes the negative samples or non-DNA-binding proteins. In this paper, we use two datasets that are extensively used in the literature for DNA-binding protein prediction problem^13,14,16,29,58^. The first dataset which we refer to as the *benchmark dataset* throughout this paper was introduced in^13^. The DNA-binding proteins were extracted from the latest version of Protein Database (PDB)^59^ with the mmCIF keyword of ‘DNA-binding protein’ using the advanced search interface. To build a high quality and non-redundance benchmark they first removed all the sequences with length less than 50 and then removed all the protein sequences with unkonwn amino acids (identified in the sequence with non-standard symbol ‘X’ or ‘Z’). Finally, they removed all the proteins with more than 25% sequence similarity using PISCES 40. In this way, they guarantee that there is no or very little structural overlap among the proteins in these benchmark^13,14,16^. As a result they build *benchmark dataset* consists of 525 DNA-binding protein and 550 non-DNA-binding protein. They specified DNA-binding and non-DNA-binding proteins in the following manner. They first specified proteins from different domains and label the one with DNA-binding sites as DNA-binding proteins and those without such sites as non-DNA-binding proteins^13,14^. Note that the input for this benchmark is a protein and not a binding domains and the target is to find if a given protein has any binding sites which is referred DNA-binding protein or not which is referred non-DNA-binding protein. It is important to highlight that having proteins with very low sequential similarity (less than 25%) with at least 50 amino acids and no unknown residue guarantee no or very low domain overlap^13,14,16,29,58^.

The second benchmark which is used as the Independent test dataset is also constructed by Lou *et al*.^15^. We use this data set wihch is referred PDB186 to be able to directly compare our results with previous studies found in the literature on an independent test set. In the dataset, 93 proteins are DNA-binding proteins and 93 proteins are non-DNA-binding proteins. They use similar strict critera to extract this benchmark as well. They first removed proteins with less than 60 amino acid length and removed those with unknown (‘X’ or ‘Z’) residue. They then used the NCBI’s BLASTCLUST^53^ to remove those proteins from the dataset that have more than 25% sequence identity.

### Feature Extraction

Different types of feature extraction methods are used in the literature of DNA-binding protein prediction. These include: pseudo position specific scoring matrix based features^16^, pseudo amino acid composition proposed by Chou and physicochemical distance transformation^29^, etc. In this study, we explore evolutionary and structural information embedded in the protein sequences as features. Protein sequences are used to fetch evolutionary information extracted as PSSM (Position-Specific Scoring Matrix) files generated by PSI-BLAST^53^. In addition to that, structural information are extracted from the spd files, output of SPIDER2^54^ software. Following sections describes the feature extraction in detail.

#### PSSM based features

We used evolutionary information from PSSM files generated using three iterations of the PSI-BLAST algorithm^53^ using the non-redundant database (nr) provided by NCBI. The cut-off threshold value of *E* was set to 0.001. PSSM file returns the log-odds of the substitution probabilities of a given protein at each position for all possible amino acid symbols after the alignment^60^. This is a *L* × 20 matrix which we refer in this paper as *PSSM matrix*. Given a protein sequence *P* consisting *L* amino acid residues as following:

2 $$P={R}_{1}{R}_{2}{R}_{3}\ldots \ldots \ldots \ldots \ldots .{R}_{L}$$

The frequency profile to *P* generated by the PSI-BLAST^53^ and matrix M can be represented as:

3 $${\mathbb{M}}=\{\begin{array}{cccc}{m}_{1,1} & {m}_{1,2} & \cdots  & {m}_{1,L}\\ {m}_{2,1} & {m}_{2,2} & \cdots  & {m}_{1,L}\\ \vdots  & \vdots  & \ddots  & \vdots \\ {m}_{20,1} & {m}_{20,2} & \cdots  & {m}_{20,L}\end{array}\}$$

where 20 is the number of standard amino acids; *m*_*i*,*j*_ is the target frequency representing the probability of amino acid i (i = 1, 2, …, 20) appearing in sequence position j (j = 1, 2, 3, … L) of protein P during evolutionary process. We first normalize the pssm matrix using the procedure proposed in^61^ for protein sub-cellular localization. After normalization, we generated five groups of features from the normalized PSSM matrix. We will denote the normalized matrix throughout this section as *N* which is a two dimensional matrix of dimension *L* × 20. The features generated from PSSM file information are enumerated in the following:

1. **Amino acid composition**: The PSSM file is used to generate a consensus sequence. A consensus sequence is built by taking the amino acid with highest substitution probability or frequency in the PSSM matrix at each position. Amino Acid composition then counts the occurrences of each amino acid residue and normalizes by the length of the protein sequence.
  4 $$AA{C}_{j}=\frac{1}{L}\,\sum _{i=1}^{L}\,aa(i,j),1\le i\le 20$$
  Here,
  $$aa(i,j)=\{\begin{array}{ll}1, & {\rm{if}}\,{s}_{j}={a}_{i}\\ 0, & {\rm{else}}\end{array}$$
  where *s*_*j*_ is an amino acid in the protein sequence and *a*_*i*_ is one of the 20 different amino acid symbols^62^.
2. **Dubchak features:** Theses features were previously used for protein fold recognition^63^ and protein subcellular localization^61^. They group the amino acid residues according to various physicochemical properties polarity, solvability, hydro-phobicity etc and calculates the composition, transition and distribution of these groupings. The size of the feature vector is 105.
3. **PSSM Bigram:** PSSM bigram represents the transition probabilities of two adjacent amino acid residue positions. These features are previously used in solving protein subcellular localization and protein fold recognition^61,63^ and defined as below:
  5 $$\mathrm{PSSM} \mbox{-} \mathrm{bigram}(k,l)=\frac{1}{L}\,\sum _{i=1}^{L-1}\,{N}_{i,k}{N}_{i+\mathrm{1,}l}\mathrm{(1}\le k\le 20,1\le l\le 20)$$
4. **PSSM 1**-**lead Bigram:** PSSM 1-lead bigram is defined as the transition probabilities of the amino acid residue positions at 1 distance or separation. It can be formally defined as:
  6 $$\mathrm{PSSM} \mbox{-} 1 \mbox{-} \mathrm{lead} \mbox{-} \mathrm{bigram}(k,l)=\frac{1}{L}\,\sum _{i=1}^{L-2}\,{N}_{i,k}{N}_{i+\mathrm{2,}l}\mathrm{(1}\le k\le 20,1\le l\le \mathrm{20)}$$
5. **PSSM Composition:** PSSM composition is created by taking the normalized sum of the values in each of the columns of the PSSM matrix^61^. Each column of the PSSM matrix represents one of the 20 amino acid residues. It is defined as:
  7 $$PSSM \mbox{-} Composition(k,l)=\frac{1}{L}\,\sum _{i=1}^{L-1}\,{N}_{i,j}\mathrm{(1}\le j\le \mathrm{20)}$$
6. **PSSM Auto**-**Covariance:** Auto-Covariance of PSSM is a feature^61,64^ depending of a distance factor, DF as parameter. In this study we used, DF = 10. The feature is formally defined as:
  8 $$\mathrm{PSSM} \mbox{-} \mathrm{Auto} \mbox{-} \mathrm{Covariance}(k,j)=\frac{1}{L}\,\sum _{i=1}^{L-k}\,{N}_{i,j}{N}_{i+k,j}\mathrm{(1}\le j\le 20,1\le k\le DF)$$
7. **PSSM Segmented Distribution:** Previously, the segmented distribution of the PSSM matrix proposed in^65^ was used as feature for sub-cellular localization of proteins in^66^. The idea is to find the distribution of the values in the PSSM matrix column wise by calculating the partial sums columnwise starting from the first row and the last row and iterating until the partial running sum is *F*_*p*_ % of the total sum. The details of the procedure for this feature generation can be found in^65–67^. In this paper, we used *F*_*p*_ = 5, 10, 25.

#### SPIDER based features

We used SPIDER2^54^, a freely available software that provides information on accessible surface area, torsion angles, structure motifs in each amino acid residue position. We then extract a novel set of features from the information provided by SPIDER2 as SPD file. The feature extraction is enumerated here in details:

1. **Secondary Structure Occurence:** There are three types of motifs structural motifs in proteins: *α*-helix (H), *β*-sheet (E) and random coil (C). Secondary Structure Occurrence is the count or frequency of each type present in mino-acid residue positions.
  9 $$\mathrm{SS} \mbox{-} \mathrm{Occurence}(i)=\sum _{j=1}^{L}\,s{m}_{ij},1\le i\le 3$$
  Here, *L* is the length of the protein and
  $$s{m}_{ij}=\{\begin{array}{ll}1, & {\rm{if}}\,S{S}_{j}={\mu }_{i}\\ 0, & {\rm{else}}\end{array}$$
  where *SS*_*j*_ is the structural motif at position *j* of the protein sequence and *μ*_*i*_ is one of the 3 different motif symbols.
2. **Secondary Structure Composition:** This feature is secondary structure motif occurrence normalized by the length of the phage protein length. This is similar to the amino acid composition except that here we are taking the count of motif symbols in stead of amino acid symbols.
  10 $$\mathrm{SS} \mbox{-} \mathrm{Occurence}(i)=\frac{1}{L}\,\sum _{j=1}^{L}\,s{m}_{ij},1\le i\le 3$$
  Here, *L* is the length of the protein and
  $$s{m}_{ij}=\{\begin{array}{ll}1, & {\rm{if}}\,S{S}_{j}={\mu }_{i}\\ 0, & {\rm{else}}\end{array}$$
  where *SS*_*j*_ is the structural motif at position *j* of the protein sequence and *μ*_*i*_ is one of the 3 different motif symbols.
3. **Accessible Surface Area Composition:** The accessible surface area composition is the normalized sum of accessible surface area defined by:
  11 $$\mathrm{ASA} \mbox{-} \mathrm{Composition}=\frac{1}{L}\,\sum _{i=1}^{L}\,ASA(i)$$
4. **Torsional Angles Composition:** For four different types of torsional angles: *ϕ*, *ψ*, *τ* and *θ* we first convert each of them into radians from degree angles and then take sign and cosine of the angles at each residue position. Thus we get a matrix of dimension *L* × 8. We denote this matrix by *T* is this section for torsional angles. Torsional angles composition is defined as:
  12 $$\mathrm{Torsional} \mbox{-} \mathrm{Angles} \mbox{-} \mathrm{Composition}({\rm{k}})=\frac{1}{L}\,\sum _{i=1}^{L}\,{T}_{i,k}\mathrm{(1}\le k\le \mathrm{8)}$$
5. **Structural Probabilities Composition:** Structural probabilities for each position of the amino acid residue are given in spd3 file as a matrix of dimension *L* × 3. We denote it by *P*. Structural probabilities composition is defined as:
  13 $$\mathrm{Structural} \mbox{-} \mathrm{Probabilities} \mbox{-} \mathrm{Composition}(k)=\frac{1}{L}\,\sum _{i=1}^{L}\,{P}_{i,k}\mathrm{(1}\le k\le \mathrm{3)}$$
6. **Torsional Angles Bigram:** Bigram for the torsional angles is similar to that of PSSM matrix and defined as:
  14 $$\mathrm{Torional} \mbox{-} \mathrm{angles} \mbox{-} \mathrm{bigram}(k,l)=\frac{1}{L}\,\sum _{i=1}^{L-1}\,{T}_{i,k}{T}_{i+\mathrm{1,}l}\mathrm{(1}\le k\le 8,1\le l\le \mathrm{8)}$$
7. **Structural Probablities Bigram:** Bigram of the structural probabilities is similar to that of PSSM matrix and defined as:
  15 $$\mathrm{Structural} \mbox{-} \mathrm{Probabilities} \mbox{-} \mathrm{bigram}(k,l)=\frac{1}{L}\,\sum _{i=1}^{L-1}\,{P}_{i,k}{P}_{i+\mathrm{1,}l}\mathrm{(1}\le k\le 3,1\le l\le \mathrm{3)}$$
8. **Torsional Angles Auto**-**Covariance:** This feature is also derived from torsional angles and defined as:
  16 $$\mathrm{Torsional} \mbox{-} \mathrm{Angles} \mbox{-} \mathrm{Auto} \mbox{-} \mathrm{Covariance}(k,j)=\frac{1}{L}\,\sum _{i=1}^{L-k}\,{T}_{i,j}{T}_{i+k,j}\mathrm{(1}\le j\le 8,1\le k\le DF)$$
9. **Structural Probablities Auto**-**Covariance:** This feature is also derived from structural probabilities and defined as:

17 $$\mathrm{Structural} \mbox{-} \mathrm{Probabilities} \mbox{-} \mathrm{Auto} \mbox{-} \mathrm{Covariance}(k,j)=\frac{1}{L}\,\sum _{i=1}^{L-k}\,{P}_{i,j}{P}_{i+k,j}\mathrm{(1}\le j\le 3,1\le k\le DF)$$

The features generated and used in this paper are summarized in Table 5.

**Table 5:** Summary of evolutionary and structural features used in this paper.

Feature Name	Feature Type	Feature Vector Size
Amino acid composition	Evolutionay(PSSM)	20
Dubchak feature	Evolutionay(PSSM)	105
Bigram	Evolutionay(PSSM)	400
PSSM composition	Evolutionay(PSSM)	20
PSSM auto covariance	Evolutionay(PSSM)	200
One lead bigram	Evolutionay(PSSM)	400
Segmented distribution	Evolutionay(PSSM)	200
Secondary structure composition	Structural(SPD3)	3
Secondary structure occurrence	Structural(SPD3)	3
ASA, Angle occurrence, probability of CHE	Structural(SPD3)	12
Bigram of angle sine cosine	Structural(SPD3)	64
Angles auto covariance	Structural(SPD3)	80
Bigram probabilities	Structural(SPD3)	9
Probabilities auto covariance	Structural(SPD3)	30

### Feature Selection

As the number of features extracted is large, we apply feature reduction to derive an optimal set of features for DNA-binding protein prediction. Previously several feature elimination techniques like correlation-based feature subset selection method^25^, tree-based feature selection^15^, best-first greedy feature selection^15^, etc. In this paper, we have used Recursive feature elimination (RFE) first proposed in^68^. The algorithm in depicted as pseudo-code in Algorithm 1. This algorithm uses backward correlation based feature elimination technique. This algorithm starts with a dataset ${\mathbb{D}}$, a classifier ${\mathbb{C}}$ and *k* the number of reduced features as parameter. In each iteration of the algorithm, the dataset is used to train a model, ${\mathbb{M}}$ and based on that the lowest ranked feature is removed. The dataset is then transformed using the resulting features. This process is continues until the number of features is equal to *k*.

**Algorithm 1:**
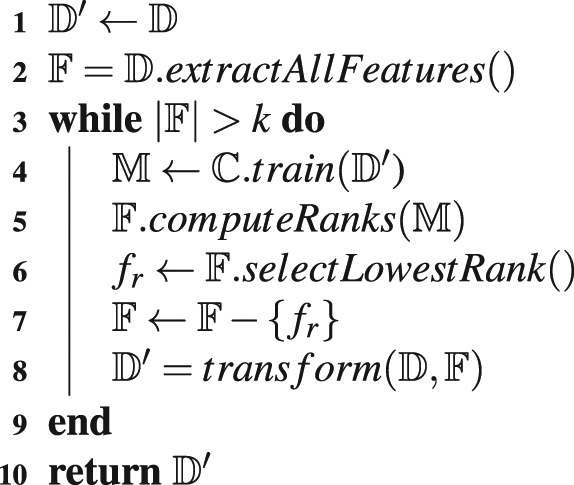
RecursiveFeatureElimination($${\mathbb{D}}$$, $${\mathbb{C}}$$, *k*).

### Description of the Classifier

We have used Support vector machine (SVM) as the classifier in our method, iDNAProt-ES. SVM^69,70^ construct a separating hyper-plane to maximize the margin between the positive and negative instances. The nearest points in the hyper-plane are called support vectors. SVM first constructs a hyper-plane based on the training dataset, and then maps an input vector from the input space into a vector in a higher dimensional space, where the mapping is determined by a kernel function. A trained SVM can output a class label (in our case, DNA-binding protein or non-DNA-binding protein) based on the mapping vector of the input vector. There are a number of popular kernels. In this paper we explore three kernel functions as described below:

1. The Linear kernel function can be defined as
  18 $$K({X}_{i},{X}_{j})={X}_{i}{X}_{j}$$
2. The (Gaussian) or Radial Basis Function kernel (RBF) can be defined as
  19 $$K({X}_{i},{X}_{j})=exp(-\gamma {(\Vert {X}_{i}-{X}_{j}\Vert )}^{2})$$
3. The Sigmoid kernel function can be defined as

20 $$K({X}_{i},{X}_{j})=\,\tanh (\gamma \mathrm{.}{X}_{i}{X}_{j})+r)$$

Here gamma and r are the kernel parameters.gamma must be greater than 0. The best kernel was the linear kernel with the parameters, *C* = 1000 and *γ* = 0.01.

### Performance Evaluation

Evaluating the performance of a new predictor is very essential^71^. Various comparison metrics are used in the literature^14,61,72^ to evaluate the performance of the predictor. There are two cross validation methods are often used: sub-sampling or K-fold(such as 5 fold, 10 fold) test and Jackknife test^73^. According to the penetrating analysis in^31^, the jackknife test is the least arbitrary than the sub-sampling test. Therefore, the jackknife test has been widely recognized and increasingly adopted by researchers to examine the quality of various predictors^74–77^ and in the literature of DNA-binding protein prediction^13,15,29,58^. In this study, we used both test K-fold cross validation and jackknife test.

We use four performance metrics, i.e. sensitivity (Sn), specificity (Sp), accuracy (Acc), Matthews correlation coefficient (MCC) and the area under the ROC curve (AUC) to measure the prediction performance as compared to the other methods in the literature. The first four metrics are defined as follows:

21 $$Sn=\frac{TP}{TP+FN}$$

22 $$Sp=\frac{TN}{TN+FP}$$

23 $$Acc=\frac{TP+TN}{TP+TN+FP+FN}$$

24 $$MCC=\frac{(TP\times TN)-(FP\times FN)}{\sqrt{(TP+FP)\,(TP+FN)\,(TN+FP)\,(TN+FN)}}$$

where TP, FP, TN and FN represent the numbers of true positives, false positives, true negatives and false negatives, respectively. The set of metrics is valid only for the single-label systems. For the multi-label systems whose existence has become more frequent in system biology^78,79^ and system medicine^41,55^, a completely different set of metrics as defined in^80^ is needed. In this study, we also use the metrics receiver-operating characteristic curve (auROC) to assess the prediction performance. Its plots the true positive rate (sensitivity) against the false positive rate (1-specificity) at different threshold settings. A predictor with perfect classification has a ROC curve passing through the top left corner (100% sensitivity and 100% specificity). Therefore, the closer the ROC curve is to the top left corner, the better the overall performance of the predictor is. Thus, auROC is used as the primary measure to assess how well a predictor can distinguish between two classes.

### Data and Material Availability

All the data and materials used in this paper are available at: http://brl.uiu.ac.bd/iDNAProt-ES/.

## Conclusion

In this paper, we present iDNAProt-ES, a novel prediction method for identification of DNA-binding proteins. We have used evolutionary and structural features for the classification extracted from PSSM files and SPD files generated by PSI-BLAST and SPIDER2, respectively. We also used recursive feature elimination to select an optimal set of features. The final model for prediction was developed using Support Vector Machine (SVM) with linear kernel. iDNAProt-ES was tested on a standard benchmark dataset and an independent dataset and achieved significantly improved results on both of the datasets. The method is freely available for use at: http://brl.uiu.ac.bd/iDNAProt-ES/.

The superiority of iDNAProt-ES was clearly noticeable in the experiments done in this study. In future, we wish to update the prediction method by incorporating an enhanced dataset. For practical application, as pointed out previously^21^, a key issue is that the number of non-DNA-binding proteins are much higher than that of DNA-binding proteins. Therefore, an enhanced dataset with balancing methods could further enhance the performance of the predictor.

## Electronic supplementary material

Supplementary Information 1

## References

[1] Lilley, D. M. *J*. *DNA*-*protein*: *structural interactions*, vol. 7 (Oxford University Press, USA, 1995).

[2] Zimmer C, Wähnert U (1986). Nonintercalating dna-binding ligands: specificity of the interaction and their use as tools in biophysical, biochemical and biological investigations of the genetic material. Prog. biophysics molecular biology.

[3] Helwa R, Hoheisel JD (2010). Analysis of dna–protein interactions: from nitrocellulose filter binding assays to microarray studies. Anal. bioanalytical chemistry.

[4] Freeman K, Gwadz M, Shore D (1995). Molecular and genetic analysis of the toxic effect of rap1 overexpression in yeast. Genet..

[5] Jaiswal R, Singh SK, Bastia D, Escalante CR (2015). Crystallization and preliminary x-ray characterization of the eukaryotic replication terminator reb1–ter dna complex. Acta Crystallogr. Sect. F: Struct. Biol. Commun..

[6] Buck MJ, Lieb JD (2004). Chip-chip: considerations for the design, analysis, and application of genome-wide chromatin immunoprecipitation experiments. Genomics.

[7] Cockerham R (1993). Nmr structure of a specific dna complex of zn-containing dna binding domain of gata-1. Sci..

[8] Douglas SM, Chou JJ, Shih WM (2007). Dna-nanotube-induced alignment of membrane proteins for nmr structure determination. Proc. Natl. Acad. Sci..

[9] Langlois RE, Lu H (2010). Boosting the prediction and understanding of dna-binding domains from sequence. Nucleic acids research.

[10] Consortium U (2017). Uniprot: the universal protein knowledgebase. Nucleic acids research.

[11] Zhao H, Yang Y, Zhou Y (2010). Structure-based prediction of dna-binding proteins by structural alignment and a volume-fraction corrected dfire-based energy function. Bioinforma.

[12] Gao M, Skolnick J (2009). A threading-based method for the prediction of dna-binding proteins with application to the human genome. PLoS Comput. Biol.

[13] Liu B (2014). Idna–prot dis: identifying dna-binding proteins by incorporating amino acid distance-pairs and reduced alphabet profile into the general pseudo amino acid composition. PloS one.

[14] Liu B, Wang S, Wang X (2015). Dna binding protein identification by combining pseudo amino acid composition and profile-based protein representation. Sci. reports.

[15] Lou W (2014). Sequence based prediction of dna-binding proteins based on hybrid feature selection using random forest and gaussian naive bayes. PLoS One.

[16] Wei L, Tang J, Zou Q (2017). Local-dpp: An improved dna-binding protein prediction method by exploring local evolutionary information. Inf. Sci.

[17] Xu R (2015). Identification of dna-binding proteins by incorporating evolutionary information into pseudo amino acid composition via the top-n-gram approach. J. Biomol. Struct. Dyn..

[18] Fang Y, Guo Y, Feng Y, Li M (2008). Predicting dna-binding proteins: approached from chou’s pseudo amino acid composition and other specific sequence features. Amino acids.

[19] Zhao X-W, Li X-T, Ma Z-Q, Yin M-H (2012). Identify dna-binding proteins with optimal chou’s amino acid composition. Protein peptide letters.

[20] Shanahan HP, Garcia MA, Jones S, Thornton JM (2004). Identifying dna-binding proteins using structural motifs and the electrostatic potential. Nucleic Acids Res.

[21] Gao M, Skolnick J (2008). Dbd-hunter: a knowledge-based method for the prediction of dna–protein interactions. Nucleic acids research.

[22] Nimrod G, Schushan M, Szilágyi A, Leslie C, Ben-Tal N (2010). Idbps: a web server for the identification of dna binding proteins. Bioinforma.

[23] Zhang Y, Arakaki AK, Skolnick J (2005). Tasser: an automated method for the prediction of protein tertiary structures in casp6. Proteins: Struct. Funct. Bioinforma..

[24] Szilágyi A, Skolnick J (2006). Efficient prediction of nucleic acid binding function from low-resolution protein structures. J. molecular biology.

[25] Kumar KK, Pugalenthi G, Suganthan P (2009). Dna-prot: identification of dna binding proteins from protein sequence information using random forest. J. Biomol. Struct. Dyn..

[26] Lin W-Z, Fang J-A, Xiao X, Chou K-C (2011). Idna-prot: identification of dna binding proteins using random forest with grey model. PloS one.

[27] Ahmad S, Gromiha MM, Sarai A (2004). Analysis and prediction of dna-binding proteins and their binding residues based on composition, sequence and structural information. Bioinforma..

[28] Kumar M, Gromiha MM, Raghava GP (2007). Identification of dna-binding proteins using support vector machines and evolutionary profiles. BMC bioinformatics.

[29] Liu B (2015). Psedna-pro: Dna-binding protein identification by combining chou’s pseaac and physicochemical distance transformation. Mol. Informatics.

[30] Dong, Q., Wang, S., Wang, K., Liu, X. & Liu, B. Identification of dna-binding proteins by auto-cross covariance transformation. In *Bioinformatics and Biomedicine* (*BIBM*), *2015 IEEE International Conference on*, 470–475 (IEEE, 2015).

[31] Chou K-C (2011). Some remarks on protein attribute prediction and pseudo amino acid composition. J. theoretical biology.

[32] Xu R (2015). Identifying dna-binding proteins by combining support vector machine and pssm distance transformation. BMC systems biology.

[33] Im J (2015). Pnimodeler: web server for inferring protein-binding nucleotides from sequence data. BMC genomics.

[34] Zhou, J., Lu, Q., Xu, R., Gui, L. & Wang, H. Cnnsite: Prediction of dna-binding residues in proteins using convolutional neural network with sequence features. In *Bioinformatics and Biomedicine* (*BIBM*), *2016 IEEE International Conference on*, 78–85 (IEEE, 2016).

[35] Paz I, Kligun E, Bengad B, Mandel-Gutfreund Y (2016). Bindup: a web server for non-homology-based prediction of dna and rna binding proteins. Nucleic acids research.

[36] Chou K-C (2015). Impacts of bioinformatics to medicinal chemistry. Medicinal chemistry.

[37] Chou K-C (2001). Prediction of protein cellular attributes using pseudo-amino acid composition. Proteins: Struct. Funct. Bioinforma.

[38] Liu B, Wu H, Chou K-C (2017). Pse-in-one 2.0: An improved package of web servers for generating various modes of pseudo components of dna, rna, and protein sequences. Nat. Sci.

[39] Chou K-C (2017). An unprecedented revolution in medicinal chemistry driven by the progress of biological science. Curr. topics medicinal chemistry.

[40] Liu B (2015). Pse-in-one: a web server for generating various modes of pseudo components of dna, rna, and protein sequences. Nucleic acids research.

[41] Cheng X, Zhao S-G, Xiao X, Chou K-C (2016). Iatc-misf: a multi-label classifier for predicting the classes of anatomical therapeutic chemicals. Bioinforma..

[42] Liu B, Wang S, Long R, Chou K-C (2016). Irspot-el: identify recombination spots with an ensemble learning approach. Bioinforma..

[43] Liu L-M, Xu Y, Chou K-C (2017). Ipgk-pseaac: identify lysine phosphoglycerylation sites in proteins by incorporating four different tiers of amino acid pairwise coupling information into the general pseaac. Medicinal Chem..

[44] Qiu, W. *et al*. Irna-2methyl: identify rna 2′-o-methylation sites by incorporating sequence-coupled effects into general pseknc and ensemble classifier. *Medicinal chemistry* (*Shariqah* (*United Arab*. *Emir*., 10.2174/1573406413666170623082245 (2017). 10.2174/1573406413666170623082245 28641529

[45] Xu Y, Wang Z, Li C, Chou K-C (2017). Ipreny-pseaac: identify c-terminal cysteine prenylation sites in proteins by incorporating two tiers of sequence couplings into pseaac. Medicinal Chem.

[46] Feng P (2017). Irna-psecoll: Identifying the occurrence sites of different rna modifications by incorporating collective effects of nucleotides into pseknc. Mol. Ther. Acids.

[47] Liu B, Yang F, Chou K-C (2017). 2l-pirna: A two-layer ensemble classifier for identifying piwi-interacting rnas and their function. Mol. Ther. Acids.

[48] Chen W (2017). Irna-ai: identifying the adenosine to inosine editing sites in rna sequences. Oncotarget.

[49] Pedregosa F (2011). Scikit-learn: Machine learning in python. J. Mach. Learn. Res..

[50] Deng, H. & Runger, G. Feature selection via regularized trees. In *Neural Networks* (*IJCNN*), *The 2012 International Joint Conference on*, 1–8, 10.1109/IJCNN.2012.6252640 (IEEE, 2012).

[51] Meinshausen N, Bühlmann P (2010). Stability selection. J. Royal Stat. Soc. Ser. B (Statistical Methodol..

[52] Bach, F. Model-consistent sparse estimation through the bootstrap. *arXiv preprint arXiv*:*0901*.*3202* (2009).

[53] Altschul SF (1997). Gapped blast and psi-blast: a new generation of protein database search programs. Nucleic acids research.

[54] Yang, Y. *et al*. Spider2: A package to predict secondary structure, accessible surface area, and main-chain torsional angles by deep neural networks. *Predict*. *Protein Second*. *Struct*. 55–63 (2017). 10.1007/978-1-4939-6406-2_6 27787820

[55] Qiu W-R, Sun B-Q, Xiao X, Xu Z-C, Chou K-C (2016). Iptm-mlys: identifying multiple lysine ptm sites and their different types. Bioinforma..

[56] Heffernan, R. *et al*. Improving prediction of secondary structure, local backbone angles, and solvent accessible surface area of proteins by iterative deep learning. *Sci*. *reports* **5**, 10.1038/srep11476 (2015). 10.1038/srep11476 PMC4476419 26098304

[57] Wang, J. *et al*. Possum: a bioinformatics toolkit for generating numerical sequence feature descriptors based on pssm profiles. *Bioinforma*., 10.1093/bioinformatics/btx302 (2017). 10.1093/bioinformatics/btx302 28903538

[58] Liu B (2015). Identification of real microrna precursors with a pseudo structure status composition approach. PloS one.

[59] Berman, H. M. *et al*. The protein data bank, 1999–. In *International Tables for Crystallography Volume F*: *Crystallography of biological macromolecules*, 675–684 (Springer, 2006).

[60] Chou K-C, Shen H-B (2007). Recent progress in protein subcellular location prediction. Anal. biochemistry.

[61] Sharma R (2015). Predict gram-positive and gram-negative subcellular localization via incorporating evolutionary information and physicochemical features into chou’s general pseaac. IEEE Transactions on NanoBioscience.

[62] Dehzangi A, Sharma A, Lyons J, Paliwal KK, Sattar A (2014). A mixture of physicochemical and evolutionary–based feature extraction approaches for protein fold recognition. Int. journal data mining bioinformatics.

[63] Sharma A, Lyons J, Dehzangi A, Paliwal KK (2013). A feature extraction technique using bi-gram probabilities of position specific scoring matrix for protein fold recognition. J. theoretical biology.

[64] Dehzangi A, Paliwal K, Lyons J, Sharma A, Sattar A (2014). A segmentation-based method to extract structural and evolutionary features for protein fold recognition. IEEE/ACM Transactions on Comput. Biol. Bioinforma..

[65] Dehzangi, A. & Sattar, A. Protein fold recognition using segmentation-based feature extraction model. In *Asian Conference on Intelligent Information and Database Systems*, 345–354 (Springer, 2013).

[66] Dehzangi A (2015). Gram-positive and gram-negative subcellular localization using rotation forest and physicochemical-based features. BMC bioinformatics.

[67] Dehzangi, A., Paliwal, K., Lyons, J., Sharma, A. & Sattar, A. Enhancing protein fold prediction accuracy using evolutionary and structural features. In *IAPR International Conference on Pattern Recognition in Bioinformatics*, 196–207 (Springer, 2013).

[68] Guyon I, Weston J, Barnhill S, Vapnik V (2002). Gene selection for cancer classification using support vector machines. Mach. learning.

[69] Cortes C, Vapnik V (1995). Support-vector networks. Mach. learning.

[70] Vapnik, V. N. & Vapnik, V. *Statistical learning theory*, vol. 1 (Wiley New York, 1998).

[71] Powers DM (2011). Evaluation: from precision, recall and f-measure to roc, informedness, markedness and correlation. J. Mach. Learn. Technol..

[72] Ding H (2016). Predicting bacteriophage proteins located in host cell with feature selection technique. Comput. biology medicine.

[73] Efron B, Gong G (1983). A leisurely look at the bootstrap, the jackknife, and cross-validation. The Am. Stat..

[74] Zeng Y-H (2009). Using the augmented chou’s pseudo amino acid composition for predicting protein submitochondria locations based on auto covariance approach. J. theoretical biology.

[75] Chang T-H (2013). Euloc: a web-server for accurately predict protein subcellular localization in eukaryotes by incorporating various features of sequence segments into the general form of chou’s pseaac. J. computer-aided molecular design.

[76] Hajisharifi Z, Piryaiee M, Beigi MM, Behbahani M, Mohabatkar H (2014). Predicting anticancer peptides with chou’s pseudo amino acid composition and investigating their mutagenicity via ames test. J. Theor. Biol..

[77] Chen Y-K, Li K-B (2013). Predicting membrane protein types by incorporating protein topology, domains, signal peptides, and physicochemical properties into the general form of chou’s pseudo amino acid composition. J. Theor. Biol..

[78] Chou K-C, Wu Z-C, Xiao X (2012). Iloc-hum: using the accumulation-label scale to predict subcellular locations of human proteins with both single and multiple sites. Mol. Biosyst..

[79] Cheng X, Xiao X, Chou K-C (2017). Ploc-mplant: predict subcellular localization of multi-location plant proteins by incorporating the optimal go information into general pseaac. Mol. BioSystems.

[80] Chou K-C (2013). Some remarks on predicting multi-label attributes in molecular biosystems. Mol. Biosyst..

