# Supplementary material for: iDNAProt-ES: Identification of DNA-binding Proteins Using Evolutionary and Structural Features
Source: Sci Rep. 2017 Nov 2;7:14938. doi: 10.1038/s41598-017-14945-1 (PMC5668250; doi:10.1038/s41598-017-14945-1)
Supplement: Supplementary file 1 — Supplementary Information 1 [file 41598_2017_14945_MOESM1_ESM.pdf]

iDNAProt-ES: Identification of DNA-binding Proteins Using Evolutionary and Structural Features

Shahana Yasmin Chowdhury[1,*], **Swakkhar Shatabda**[1], **and Abdollah Dehzangi**[2]
[1]Department of Computer Science and Engineering, United International University, House 80, Road 8A, Dhanmondi, Dhaka-1209, Bangladesh
[2]Department of Computer Science, Morgan State University, Baltimore, Maryland, United States
[*]Correspondence to: swakkhar@cse.uiu.ac.bd

Supporting Information # 1

List of features selected by the recursive feature elimination technique from all the combined evolutionary and structural features. The table below contains the feature number and the feature group it comes from.

**Feature Number**	**Feature Group**
Feature_110	Dubchuck
Feature_142	PSSM Bigram
Feature_145	PSSM Bigram
Feature_146	PSSM Bigram
Feature_187	PSSM Bigram
Feature_219	PSSM Bigram
Feature_250	PSSM Bigram
Feature_264	PSSM Bigram
Feature_266	PSSM Bigram
Feature_272	PSSM Bigram
Feature_277	PSSM Bigram
Feature_279	PSSM Bigram
Feature_380	PSSM Bigram
Feature_321	PSSM Bigram
Feature_323	PSSM Bigram
Feature_324	PSSM Bigram
Feature_330	PSSM Bigram
Feature_335	PSSM Bigram
Feature_342	PSSM Bigram
Feature_346	PSSM Bigram
Feature_373	PSSM Bigram
Feature_386	PSSM Bigram
Feature_393	PSSM Bigram
Feature_427	PSSM Bigram
Feature_444	PSSM Bigram
Feature_459	PSSM Bigram
Feature_475	PSSM Bigram
Feature_479	PSSM Bigram

Feature_496	PSSM Bigram
Feature_522	PSSM Bigram
Feature_574	PSSM Auto-Covariance
Feature_577	PSSM Auto-Covariance
Feature_590	PSSM Auto-Covariance
Feature_593	PSSM Auto-Covariance
Feature_601	PSSM Auto-Covariance
Feature_610	PSSM Auto-Covariance
Feature_622	PSSM Auto-Covariance
Feature_640	PSSM Auto-Covariance
Feature_641	PSSM Auto-Covariance
Feature_666	PSSM Auto-Covariance
Feature_690	PSSM Auto-Covariance
Feature_694	PSSM Auto-Covariance
Feature_703	PSSM Auto-Covariance
Feature_714	PSSM Auto-Covariance
Feature_736	PSSM Auto-Covariance
Feature_742	PSSM Auto-Covariance
Feature_754	PSSM 1-lead Bigram
Feature_767	PSSM 1-lead Bigram
Feature_775	PSSM 1-lead Bigram
Feature_799	PSSM 1-lead Bigram
Feature_808	PSSM 1-lead Bigram
Feature_815	PSSM 1-lead Bigram
Feature_858	PSSM 1-lead Bigram
Feature_862	PSSM 1-lead Bigram
Feature_908	PSSM 1-lead Bigram
Feature_911	PSSM 1-lead Bigram
Feature_928	PSSM 1-lead Bigram
Feature_946	PSSM 1-lead Bigram
Feature_951	PSSM 1-lead Bigram
Feature_974	PSSM 1-lead Bigram
Feature_992	PSSM 1-lead Bigram
Feature_1004	PSSM 1-lead Bigram
Feature_1029	PSSM 1-lead Bigram
Feature_1040	PSSM 1-lead Bigram
Feature_1064	PSSM Segmented Distribution
Feature_1075	PSSM Segmented Distribution
Feature_1089	PSSM Segmented Distribution
Feature_1090	PSSM Segmented Distribution
Feature_1094	PSSM Segmented Distribution
Feature_1104	PSSM Segmented Distribution
Feature_1119	PSSM Segmented Distribution
Feature_1131	PSSM Segmented Distribution
Feature_1134	PSSM Segmented Distribution
Feature_1219	PSSM Segmented Distribution
Feature_1273	PSSM Segmented Distribution
Feature_1283	PSSM Segmented Distribution

Feature_1308	PSSM Segmented Distribution
Feature_1373	Angles Bigram
Feature_1376	Angles Bigram
Feature_1386	Angles Bigram
Feature_1392	Angles Bigram
Feature_1393	Angles Bigram
Feature_1486	Angles Auto-Covariance
Feature_1519	Probabilities Auto-Covariance
Feature_1528	Probabilities Auto-Covariance
Feature_1537	Probabilities Auto-Covariance
